# Development and Validation of a Nomogram to Predict Respiratory Failure With Influenza

**DOI:** 10.1002/iid3.70481

**Published:** 2026-06-25

**Authors:** Mingzhen Zhao, Yi Li, Hongxiang Fu, Guanghui Jia, Xing Zhao, Yu Sun, Xingbin Li, Hongbo Zhang, Zhiwei Zhao

**Affiliations:** ^1^ Hebei Key Laboratory of Panvascular Diseases Affiliated Hospital of Chengde Medical University Chengde Hebei China; ^2^ Clinical Medicine Department, Central Research Institute for Generic Drug Preparations Huahai Pharmaceutical Co., LTD Linhai Zhejiang China; ^3^ Department of Respiratory and Critical Care Medicine Affiliated Hospital of Chengde Medical University Chengde Hebei China; ^4^ Department of Pathology Affiliated Hospital of Chengde Medical University Chengde Hebei China; ^5^ Department of spine surgery Affiliated Hospital of Chengde Medical University Chengde Hebei China; ^6^ Department of Respiratory and Critical Care Medicine Hebei Chest Hospital Shijiazhuang Hebei China; ^7^ Department of Pathophysiology Chengde Medical University Chengde Hebei China

**Keywords:** age, influenza, influenza type, nomogram, RDW.CV, respiratory failure, tumor

## Abstract

**Purpose:**

Influenza‐induced respiratory failure is a severe complication of influenza that can rapidly progress to multi‐organ failure or even death due to severe impairment of pulmonary ventilation or gas exchange function. Early prediction and intervention can decrease the mortality in patients with respiratory failure.

**Methods:**

A comprehensive analysis was conducted on 182 influenza‐positive patients who were admitted in Affiliated Hospital of Chengde Medical University between December 2018 and May 2019. 78 patients with influenza were used for external validation at Hebei Chest Hospital. We assessed the relationship between respiratory failure and demographic characteristics, preexisting diseases, and laboratory test results in the training group. First, the variables of the nomogram of respiratory failure with influenza were selected using Least absolute shrinkage and selection operator (LASSO) and multivariate logistic regression analysis. A nomogram was developed to predict respiratory failure due to influenza. The accuracy of the proposed model was validated by utilizing the area under the receiver operating characteristic (ROC) and calibration curve. The clinical utility of the proposed model was evaluated using decision curve analysis (DCA) and clinical impact curve (CIC).

**Results:**

A total of 182 influenza‐positive patients were included, with the incidence of respiratory failure reaching 29.1% (*n* = 53). Risk factors contributing to respiratory failure encompassed age, tumor, influenza type, and red cell distribution width coefficient of variation (RDW.CV). Utilizing ROC and calibration curve assessment, the constructed nomogram exhibited accurate prediction of respiratory failure risk, and the AUC value in the training group was 0.79. Of the 78 patients diagnosed with influenza, 35 (accounting for 44.9%) developed respiratory failure. Using the ROC curve, the established nomogram accurately predicted the risk of respiratory failure, and the area under the curve was 0.73 in the validation cohort. Decision curve analysis and clinical impact curve verified that this model demonstrated excellent clinical utility in predicting respiratory failure among influenza patients.

**Conclusion:**

A nomogram based on the expression levels of RDW.CV, influenza type, tumor, and age was an efficient model for the early identification of respiratory failure in patients with influenza. These results will be useful for guiding the prevention and treatment of respiratory failure caused by influenza.

## 1 Introduction

Among the most widespread viruses, influenza viruses are key contributors to worldwide pandemics, which exert a catastrophic threat to human health [1]. There have been numerous influenza pandemics, such as the 1918 Spanish (H1N1), 1957 Asian (H2N2), 1968 Hong Kong (H3N2), and the 2009 H1N1 [2]. It is important to highlight that during pandemic periods, influenza outbreaks result in an average of 389,000 respiratory deaths each year [3]. According to previous reports, some patients with severe illnesses tend to develop viral pneumonia, acute kidney injury, myocardial injury, multiple organ failure, disseminated intravascular coagulation, respiratory failure, and other complications [4, 5, 6]. It is well known that respiratory failure is a clinical syndrome of severe disorders of pulmonary ventilation or gas exchange caused by various reasons, resulting in ineffective gas exchange, leading to hypoxemia with (or without) hypercapnia, and thus causing a series of physiological and metabolic disorders. Respiratory failure is the most important complication of the influenza virus infection and contributes to high mortality [6]. Patients with mild influenza are prone to missing opportunities for early intervention and appropriate therapeutic measures, due to the inability of physicians to predict early progression of mild influenza to respiratory failure. Therefore, to improve patient prognosis, it is essential to identify early influenza patients who are at risk of developing respiratory failure, strengthen surveillance timely, and deliver effective treatment.

As a visual predictive tool, the nomogram is capable of calculating the individual outcome risk and has been extensively applied in disease prevention evaluation in recent years [6, 7, 8, 9]. Although various studies have assessed the risk factors contributing to respiratory failure, there remains a lack of universally accepted individualized prediction nomograms for respiratory failure in patients with influenza [10, 11, 12, 13]. To enable clinicians to identify respiratory failure at an early stage and decrease its morbidity and mortality, we developed and validated a specialized nomogram for influenza‐associated respiratory failure.

## 2 Methods

### 2.1 Study Design

We screened patients diagnosed with influenza at the Affiliated Hospital of Chengde Medical University and Hebei Chest Hospital between December 2018 and May 2019. In our study, the Affiliated Hospital of Chengde Medical University was designated as the training group, and Hebei Chest Hospital as the validation group. The inclusion criterion was a diagnosis of influenza (including Type I and Type II respiratory failure). Type I respiratory failure mainly manifests as hypoxia with an oxygen partial pressure below 60 mmHg, and the carbon dioxide partial pressure can be normal or decreased. Type II respiratory failure is accompanied by hypoxia and carbon dioxide retention at the same time, with an oxygen partial pressure below 60 mmHg and a carbon dioxide partial pressure above 50 mmHg. Participants were excluded if they were pregnant or had incomplete data on preexisting conditions or laboratory test results.

### 2.2 Variables of Respiratory Failure With Influenza

The variables in our study included demographic characteristics, preexisting diseases, and laboratory tests. Demographic characteristics included sex, age, influenza type, smoking behavior, and preexisting diseases, included hypertension, diabetes mellitus, coronary heart disease, tumor, and diseases of the respiratory system, brain system, kidney system, and cardiovascular system. Routine laboratory tests included serum white blood cell (WBC) count, neutrophil ratio (NEUT%), lymphocyte ratio (LYMPH%), monocyte ratio (MONO%), and RDW.CV. First, we used univariate LASSO analysis to verify the risk factors for respiratory failure in patients with influenza in the training group. Using 10‐fold cross‐validation, the variables were centralized and normalized using the R software. Subsequently, the results from LASSO regression analysis and multiple logistic regression analysis were combined, and R software was used to derive accurate predictors.

### 2.3 Development and Validation of a Nomogram of Respiratory Failure With Influenza

A nomogram was constructed to predict respiratory failure due to influenza in the training group using the R software. This model contained four variables: age, tumor, RDW.CV and influenza type. ROC analysis was implemented to assess predictive ability of the prediction model with Hmisc and ROCR packages in the training and validation groups. Furthermore, calibration curve analysis for respiratory failure in influenza patients demonstrated favorable concordance between the model‐predicted probabilities and the clinically observed probabilities in the training and validation groups. To meet the actual needs of clinical decision‐making, decision curve analysis (DCA) and clinical impact curve (CIC) were performed using RMDA package.

## 3 Results

### 3.1 Patient Characteristics

**Figure 1:**
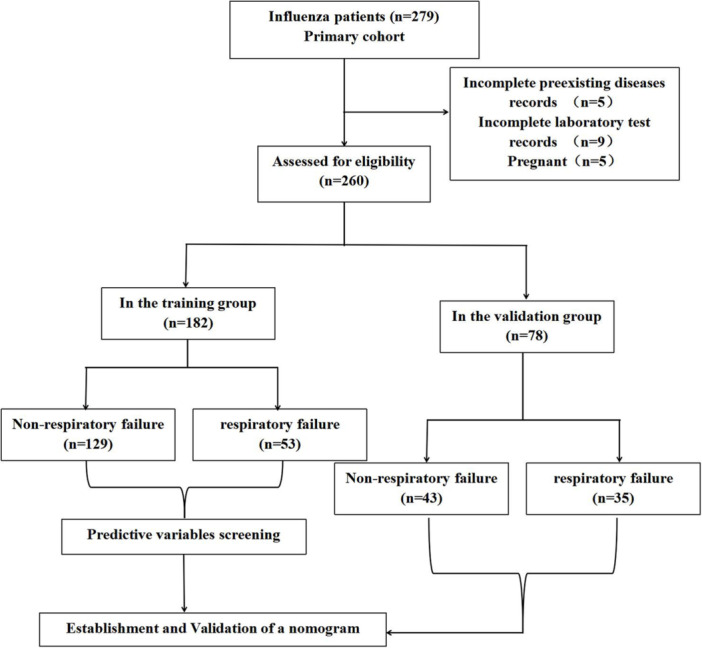
Flow diagram of data collection and analysis.

In total, 182 eligible patients were enrolled in the training group (Figure 1). Among them, 53 had respiratory failure, and 129 had non‐respiratory failure at our hospital. The median age of all the participants was 60 years (IQR, 41.25–72 years); the study included 71 (39%) females and 111 (61%) males. Patients information included demographic characteristics, preexisting diseases, and laboratory test (Table 1). Seventy‐eight eligible patients were included in the validation group. Among these patients, 35 had respiratory failure, and 43 had non‐respiratory failure at Hebei Chest Hospital. Among all the participants in the study, the median age was 67.5 years (IQR, 56–76.25 years), and 26 (33%) females and 52 (67%) males were included. Their patients information included demographic characteristics, preexisting diseases, and laboratory test (Table 2).

**Table 1:** Baseline characteristics of study patients with influenza at the Affiliated Hospital of Chengde Medical University.

Variables	Total (*n* = 182)	Respiratory failure (*n* = 53)	Non‐respiratory failure (*n* = 129)	*p*
Age, median (Q1,Q3)	60 (41.25, 72)	68 (62, 78)	54 (14, 67)	< 0.001***
Sex, *n* (%)				0.345
Female	71 (39)	24 (45)	47 (36)	
Male	111 (61)	29 (55)	82 (64)	
influenza type, *n* (%)				< 0.001***
A	149 (82)	52 (98)	97 (75)	
B	33 (18)	1 (2)	32 (25)	
Tumor, *n* (%)				0.003**
No	171 (94)	45 (85)	126 (98)	
Yes	11 (6)	8 (15)	3 (2)	
Smoking, *n* (%)				0.279
No	119 (65)	31 (58)	88 (68)	
Yes	63 (35)	22 (42)	41 (32)	
Hypertension, *n* (%)				0.721
No	122 (67)	34 (64)	88 (68)	
Yes	60 (33)	19 (36)	41 (32)	
Diabetes mellitus, *n* (%)				0.04*
No	167 (92)	45 (85)	122 (95)	
Yes	15 (8)	8 (15)	7 (5)	
coronary heart disease, *n* (%)				0.77
No	155 (85)	44 (83)	111 (86)	
Yes	27 (15)	9 (17)	18 (14)	
Diseases of the respiration system, *n* (%)				0.03*
No	141 (77)	35 (66)	106 (82)	
Yes	41 (23)	18 (34)	23 (18)	
Diseases of the brain system, *n* (%)				0.1
No	146 (80)	38 (72)	108 (84)	
Yes	36 (20)	15 (28)	21 (16)	
Diseases of the kidney system, *n* (%)				0.721
No	173 (95)	50 (94)	123 (95)	
Yes	9 (5)	3 (6)	6 (5)	
Diseases of the cardiovascular system, *n* (%)				0.769
No	167 (92)	48 (91)	119 (92)	
Yes	15 (8)	5 (9)	10 (8)	
WBC, Median (Q1,Q3)	6.25 (4.89, 9.35)	6.6 (5.25, 10.5)	6.2 (4.85, 9.24)	0.256
NEUT%, Median (Q1,Q3)	73.85 (61.15, 83.33)	82.3 (69.7, 87.8)	69.2 (58, 80.1)	< 0.001***
LYMPH%, Median (Q1,Q3)	16.25 (8.93, 27.48)	11.7 (6, 19.1)	18.7 (11.4, 29.7)	**< **0.001***
MONO%, Median (Q1,Q3)	7.4 (5, 11.3)	6.5 (4.1, 9.6)	7.5 (5.7, 11.5)	0.02*
RDW.CV, Median (Q1,Q3)	13.1 (12.5, 14.07)	13.5 (12.9, 14.9)	12.9 (12.4, 13.6)	< 0.001***

*Note:* Data presented as median or numbers, with percentages in parentheses, *p* < 0.05 considered statistically significant. Significance codes: ‘***’ 0.001, ‘**’ 0.01, ‘*’ 0.05.

Abbreviations: LYMPH%, Lymphocyte percentage; MONO%, Monocyte percentage; NEUT%, Neutrophil ratio; RDW.CV, Red cell distribution width ‐ coefficient of variation; WBC, White blood cell count.

**Table 2:** Baseline characteristics of study patients with influenza at Hebei Chest Hospital.

Variables	Total (*n* = 78)	Respiratory failure (*n* = 35)	Non‐respiratory failure (*n* = 43)	*p*
Age, median (Q1,Q3)	67.5 (56, 76.25)	71 (63, 80)	62 (49, 73)	0.009**
Sex, *n* (%)				0.295
Female	26 (33)	9 (26)	17 (40)	
Male	52 (67)	26 (74)	26 (60)	
Influenza type, *n* (%)				0.499
A	76 (97)	35 (100)	41 (95)	
B	**2 (3)**	**0 (0)**	**2 (5)**	
Tumor, *n* (%)				**1**
No	74 (95)	33 (94)	41 (95)	
Yes	4 (5)	2 (6)	2 (5)	
Smoking, *n* (%)				0.818
No	49 (63)	21 (60)	28 (65)	
Yes	29 (37)	14 (40)	15 (35)	
Hypertension, *n* (%)				0.295
No	59 (76)	24 (69)	35 (81)	
Yes	19 (24)	11 (31)	8 (19)	
Diabetes mellitus, *n* (%)				0.182
No	66 (85)	27 (77)	39 (91)	
Yes	12 (15)	8 (23)	4 (9)	
coronary heart disease, *n* (%)				0.19
No	60 (77)	24 (69)	36 (84)	
Yes	18 (23)	11 (31)	7 (16)	
Diseases of the respiration system, *n* (%)				0.925
No	35 (45)	15 (43)	20 (47)	
Yes	43 (55)	20 (57)	23 (53)	
Diseases of the brain system, *n* (%)				1
No	70 (90)	31 (89)	39 (91)	
Yes	8 (10)	4 (11)	4 (9)	
Diseases of the kidney system, *n* (%)				0.623
No	74 (95)	34 (97)	40 (93)	
Yes	4 (5)	1 (3)	3 (7)	
Diseases of the cardiovascular system, *n* (%)				0.113
No	61 (78)	24 (69)	37 (86)	
Yes	17 (22)	11 (31)	6 (14)	
WBC, median (Q1,Q3)	7.11 (4.71, 12.08)	9.63 (5.32, 12.86)	6.28 (4.68, 8.5)	0.115
NEUT%, median (Q1,Q3)	81.1 (63.55, 88.68)	87.2 (79.95, 91.8)	73 (59.8, 82.05)	< 0.001***
LYMPH%, median (Q1,Q3)	12.4 (7.05, 23.02)	8.6 (5.3, 12.2)	18.5 (10.1, 31.5)	< 0.001***
MONO%, median (Q1,Q3)	5.1 (3.05, 8.12)	4.4 (2.15, 5.9)	5.5 (4.3, 8.3)	0.03*
RDW.CV, Median (Q1,Q3)	13.25 (12.6, 14.1)	13.7 (12.95, 14.6)	13 (12.6, 13.65)	0.009**

*Note:* Data presented as median or numbers, with percentages in parentheses, *p* < 0.05 considered statistically significant. Signifance codes: ‘***’ 0.001, ‘**’ 0.01, ‘*’ 0.05.

Abbreviations: LYMPH, Lymphocyte percentage, MONO, monocyte percentage; NEUT, Neutrophil ratio; RDW.CV, Red cell distribution width ‐ coefficient of variation; WBC, White blood cell count.

### 3.2 Selected Variables for the Nomogram

**Figure 2:**
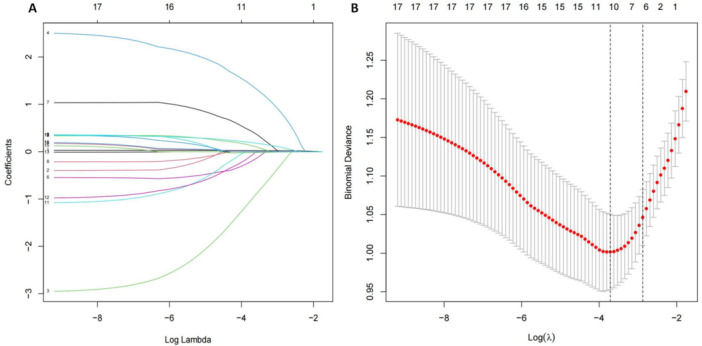
LASSO coefficient profiles of severe influenza pneumonia. Each curve in the figure presents the change of each variable in coefficient. The ordinate is the coefficient value, the lower abscissa is log(λ), and the upper abscissa is the number of non‐zero coefficients in the model at this time (A). 10‐fold cross‐validation fitting and then selecting the model (B) in the training.

To select the variables that predicted respiratory failure, demographic characteristics (sex, age, influenza type, and smoking behavior), preexisting diseases (hypertension, diabetes mellitus, coronary heart disease, diseases of the respiratory brain, kidney, and cardiovascular system, and tumor), and routine laboratory testing (WBC, NEUT%, LYMPH%, RDW.CV, and MONO%) were included in the LASSO regression analysis in the training group. Ten potential predictors were screened using LASSO regression analysis, including age, influenza type, tumor, hypertension, diabetes mellitus, respiratory system diseases, cardiovascular disease, NEUT%, LYMPH%, and RDW.CV (Figure 2). With results of LASSO as support, we carried out a subsequent multiple logistic regression analysis. We obtained four final nomogram variables: age, tumor, influenza type, and RDW.CV (Table 3).

**Table 3:** Multivariate logistic regression analysis of predictors selected by LASSO regression procedure in the dataset.

Independent variables	Multivariable logistic regression analysis	
	OR (95% CI)	*p*‐value
**Age**	**1.02971947 (1.002658 1.0610437)**	0.04127*
Influenza type	0.05839364 (0.002050 0.5512378)	0.03769*
Tumor	8.66752586 (1.784253 65.6526425)	0.01461*
Hypertension	0.49087386 (0.204530 1.1304924)	0.100960
Diabetes mellitus	2.63802382 (0.777199 9.3653648)	0.121630
Diseases of the respiration system	1.37514094 (0.558674 3.3411850)	0.482650
Diseases of the cardiovascular system	0.460225 (0.112007 1.6314947)	0.247000
NEUT%	1.002399 (0.955294 1.0597329)	0.920870
LYMPH%	0.957180 (0.891613 1.0323659)	0.216050
RDW. CV	1.406789 (1.099287 1.8197915)	0.00717**

*Note:* Significant codes: ‘**’0.01, ‘*’ 0.05.

Abbreviations: Cl, confidence interval; LASSO, least absolute shrinkage and selection operator; OR, odd ratio; RDW.CV, Red cell distribution width ‐ coefficient of variation.

#### 3.2.1 Developed a Nomogram of Respiratory Failure With Influenza

**Figure 3:**
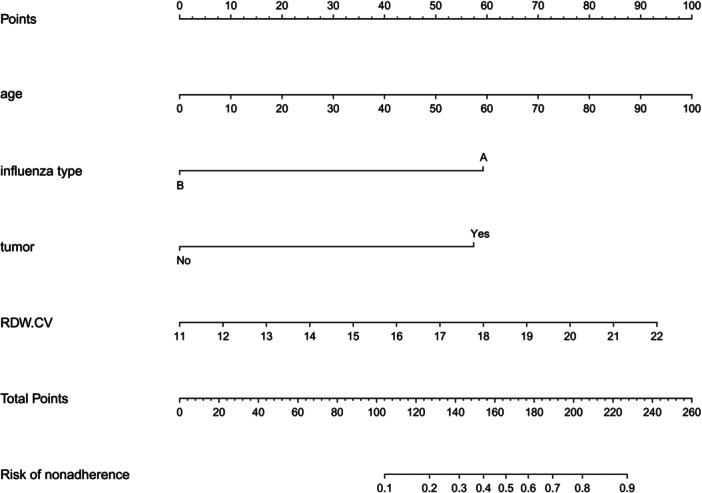
A nomogram for respiratory failure was designed with the variables. The variables points were found on the uppermost point scale that matched with each patient variable and was added. The total points extrapolated to the bottom scale show the percent probability of respiratory failure in the training.

Using the results of logistic regression analysis, a nomogram was developed to predict influenza‐induced respiratory failure based on these four variables. The “Total Points” can be calculated based on the contribution values (Points) of each predictor to the prediction results. Each total point corresponded to a corresponding risk value representing the probability of respiratory failure in each patient, as shown in Figure 3.

#### 3.2.2 Validated a Nomogram for the Probability of Respiratory Failure

**Figure 4:**
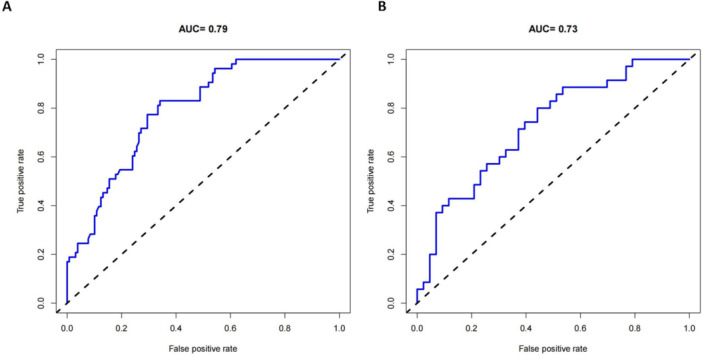
ROC of the nomogram in the datasets. The nomogram exhibited excellent power of discrimination with an AUC of 0.79 in the training. ROC, receiver operating characteristic; AUC, area under the curve in the training (A) and the area under the curve (AUC) of the nomogram was 0.73 in validation group (B).

**Figure 5:**
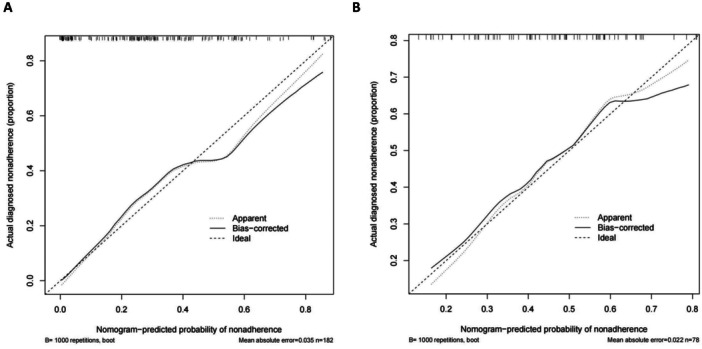
Calibration plot of Nomogram in the datasets. The x‐axis is the predicted probability of respiratory failure of influenza. The y‐axis is the observed respiratory failure of influenza. The diagonal dotted line represents a perfect prediction by an ideal model. The solid line represents the performance of the nomogram. It represents a better prediction that a solid line is close to a diagonal dotted line. The figure shows that the prediction model has a good predictive ability in the training (A) and in the validation group (B).

The consistency between the predicted probability of respiratory failure in the influenza patients using the established nomogram and the actual probability of respiratory failure was evaluated using receiver operating characteristic curve (ROC) and calibration curves. The area under the curve (AUC) of the nomogram was 0.79 in Figure 4A and 0.73 in the Figure 4B in the training group and the validation group, respectively, which indicated that the predictive model had good predictive ability. Calibration curves were used to evaluate the consistency between the predicted and actual probabilities of respiratory failure in patients with influenza. Using the bootstrap self‐sampling technique, the calibration curves of the nomogram, which were repeated 1000 times, displayed high consistency between the predicted and observed probabilities of respiratory failure in the training and validation groups (Figure 5A,B). Collectively, these results implied that the nomogram for respiratory failure is equipped with reliable and accurate calibration abilities.

#### 3.2.3 Clinical Value of the Nomogram

**Figure 6:**
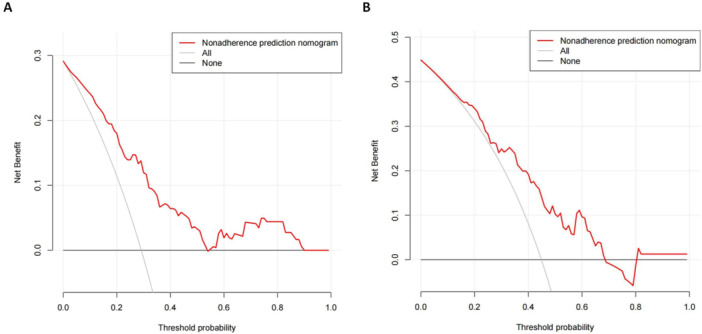
Decision curve analysis (DCA) of the nomogram in the datasets. Dark gray‐solid line: The patient does not apply the nomogram and the net beneft is zero; Light Gray‐solid line: All patients are treated by the nomogram. The area enclosed by the three lines presents the clinical utility of the nomogram in the training (A) and in the validation group (B).

**Figure 7:**
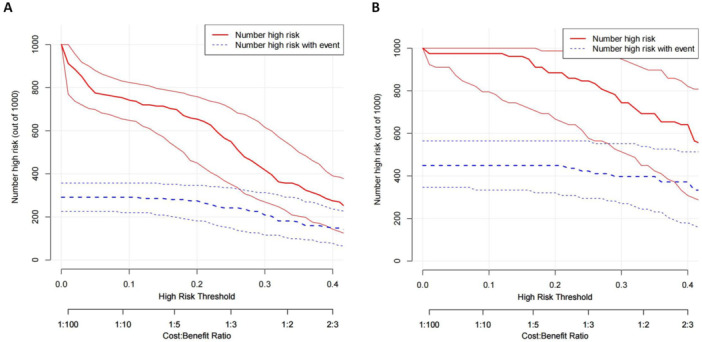
Clinical impact curve (CIC) of respiratory failure of influenza model. The red curve (number of high‐risk individuals) indicates the number of people who are classified as positive (high risk) by the model at each threshold probability; the blue curve (number of high‐risk individuals with outcome) is the number of true positives at each threshold probability. CIC visually indicated that the nomogram conferred high clinical net benefit and confirmed the clinical value of the respiratory failure of influenza model in the training (A) and in the validation group (B).

Clinical application of the nomogram was assessed by means of DCA and CIC. The DCA results indicated that, within the threshold range of 0.01–0.9 and 0.01–0.7, in the training and validation groups, using the established nomogram to predict respiratory failure in influenza patients is more advantageous than using the all patient intervention or no intervention methods (Figure 6A,B). The CIC results are shown in Figure 7A,B, which display the predicted risk range and cost‐benefit ratio of influenza patients with actual respiratory failure.

## 4 Discussion

Although patients with mild influenza can be treated, the mortality rate from respiratory failure due to influenza is relatively high. Earlier studies have confirmed that the most effective prevention strategy is the early identification of individuals who are at risk of developing respiratory failure. Nomograms can be used as visual prediction tools to estimate individual outcome risks early. They have also been used for the diagnosis and prognosis of various diseases. However, nomograms are rarely used as predictive models for influenza‐associated respiratory failure. The accurate prediction and estimation of respiratory failure in patients with influenza are essential for patient counseling and treatment decision‐making. In this study, we developed a nomogram for influenza‐associated respiratory failure. Through validation, the nomogram accurately predicted the possibility of respiratory failure in the early stages. Therefore, this can aid clinicians in enhanced decision‐making at the earliest to reduce mortality rates, and patients can gain more net benefits.

RDW.CV, which stands for red cell distribution width‐coefficient of variation, is a component of the complete blood count test, as it is used to measure the variation in the size and volume of red blood cells. A higher RDW.CV value indicates greater heterogeneity in the size of red blood cells [14]. Previous studies have reported that higher RDW.CV values are related to poor outcomes in many diseases, particularly acute appendicitis, hepatocellular carcinoma, cardiovascular disease, ischemic stroke, and so on [15, 16, 17, 18]. Although the relationship between the biological mechanisms of the RDW‐CV values and non‐hematological diseases is not yet clear, several hypotheses have been reported. Jaiswal S et al. reported that clonal hematopoiesis (CH) is a somatic mutation that occurs in the hematopoietic stem cells and increases in age‐related diseases [19]. In addition, another study showed that inflammation affects RDW.CV values by inhibiting bone marrow erythropoiesis or shortening RBC survival [20, 21]. Recently, researchers have repeatedly reported that RDW.CV values are associated with the severity, progression, and prognosis of influenza [22, 23, 24, 25]; conversely, data on influenza‐associated respiratory failure (RF) remain extremely insufficient. Our study found that a higher RDW.CV was associated with a higher incidence of respiratory failure due to influenza. Up to now, there have been no studies on RDW‐CV levels as a variable nomogram for respiratory failure with influenza.

It is widely recognized that cancer patients are susceptible to influenza viruses and face a high risk of developing severe complications (including respiratory failure) [26, 27]. During the 2009 influenza pandemic, patients diagnosed with hematological malignancies faced an elevated risk of developing influenza pneumonia and acute respiratory distress syndrome (ARDS), which ultimately led to severe respiratory failure [28]. On one hand, patients with cancer are often immunocompromised due to the consumption of the disease [29, 30], whereas they often suffer from immune dysfunction due to treatment with medicines [31, 32]. Moreover, influenza vaccination is recommended for patients with malignancies. However, documented vaccination rates are only 30–50% among patients with cancer [33]. Hence, patients with cancer are assumed to be at high risk of influenza‐related morbidity and mortality.

Influenza is an acute viral respiratory infection affecting all age groups. However, age has been reported as a risk factor associated with higher severity and lethality of influenza [9, 34]. During an epidemic, the death rates from pneumonia for the elderly are higher than that for younger people [35, 36, 37]. As reported by Miron VD, age represents a significant predictor of both hospitalization duration and acute respiratory failure in patients diagnosed with influenza; older patient age is linked to an 8.9‐fold elevated risk of acute respiratory failure. Multiple studies have confirmed that aging is associated with changes in T‐cell immunity, along with a low‐level chronic inflammatory known as inflammaging. Aging is increasingly recognized as an inherent characteristic of the pathological progression of numerous age‐related diseases, such as cardiovascular diseases, cancer, diabetes mellitus, metabolic syndrome, frailty syndrome, and chronic obstructive pulmonary disease (COPD) [16, 17, 38]. Therefore, elderly individuals with influenza are at risk of respiratory failure.

Human influenza viruses are primarily divided into three types: A, B, and C. The severity of the different types of influenza can vary [39]. Symptoms caused by B‐or C‐type influenza viruses are slightly more common than those caused by A‐type influenza. Fever, cough, and sore throat are the main symptoms, and the systemic symptoms may not be as severe as those caused by type A influenza [40, 41, 42]. One type of influenza virus includes several subtypes (H1N1, H3N2, H5N1, and H7N9). Most patients affected by influenza A virus have relatively mild symptoms like fever, cough, runny nose, sore throat, headache, and fatigue, which resemble the symptoms of patients infected with influenza B or C viruses. However, in some cases, the A‐type influenza virus can cause severe complications, such as pneumonia, respiratory failure, organ failure, and heart failure, thereby increasing the risk of death [43, 44, 45]. Overall, severe influenza is not only related to the age of the patient, basic health condition, timely treatment, influenza vaccination, and many other factors, but it also depends on the type of influenza virus. According to incomplete statistics, the most fatal influenza pandemic worldwide is type A [2].

In conclusion, we established a nomogram for predicting respiratory failure in influenza patients by leveraging influenza datasets. Based on the expression levels of RDW.CV, along with age, influenza type, and tumor status, this nomogram model has been confirmed as a dependable prediction model for respiratory failure.

While the developed prognostic nomogram possesses reliable predictive ability for respiratory failure in influenza patients, our study has several limitations. First, an inherent limitation of this study was its retrospective design. Second, the Affiliated Hospital of Chengde Medical University is a local comprehensive hospital that lacks relevant pediatric cases. Third, our sample size was relatively small. In subsequent research, more detailed and comprehensive prospective cohort studies involving larger sample sizes should be formulated to further elevate the accuracy of our predictive nomogram.

## 5 Conclusions

The nomogram of respiratory failure based on the expression levels of RDW. CV, age, influenza type, and tumor showed good discrimination, calibration, and clinical value.

## Author Contributions

**Mingzhen Zhao:** methodology, writing – original draft. **Yi Li:** methodology, software. **Hongxiang Fu:** data curation. **Guanghui Jia:** data curation. **Xing Zhao:** software. **Yu Sun:** writing – review and editing. **Xingbin Li:** data curation. **Hongbo Zhang:** conceptualization. **Zhiwei Zhao:** conceptualization, writing – review and editing.

## Ethics Statement

The Ethics Committee of the Affiliated Hospital of Chengde Medical University approved the conduct of all experiments.

## Conflicts of Interest

The authors declare no conflicts of interest.

## Data Availability

The data that support the findings of this study are available from the corresponding author upon reasonable request.
